# POTENTIALLY INAPPROPRIATE CENTRAL NERVOUS SYSTEM MEDICATION PRESCRIBING AMONG SEXUAL AND GENDER MINORITY ADULTS

**DOI:** 10.1093/geroni/igae098.1924

**Published:** 2024-12-31

**Authors:** Chelsea Wong, Louisa Smith, Robert Cavanaugh, Dae Hyun Kim, Carl Streed, Farzana Kapadia, Brianne Olivieri-Mui

**Affiliations:** Beth Israel Deaconess Medical Center, Brookline, Massachusetts, United States; Northeastern University, Boston, Massachusetts, United States; Roux Institute, Northeastern University, Portland, Maine, United States; Hebrew SeniorLife, Boston, Massachusetts, United States; Boston University School Of Medicine, Boston, Massachusetts, United States; New York University, New York, New York, United States; Northeastern University, Boston, Massachusetts, United States

## Abstract

Among older, cisgender heterosexual adults (non-OSGM) mental health disorders and frailty are associated with higher potentially inappropriate medications (PIM). Yet, this association remains unclear among older sexual and gender minority adults (OSGM) who experience higher burden of frailty at earlier ages. We aimed to assess PIM prescribing by OSGM status and frailty. Using the All of Us Database (v7, Controlled Tier), we included participants ≥50yo without a diagnosis for appropriate use of four central nervous system PIM classes: hypnotics /sedatives, antipsychotics, antidepressants, or anxiolytics. Separate logistic regression models for each PIM category were constructed to estimate the association of OSGM status and frailty category with PIM prescriptions adjusting for age, race and ethnicity, income, marital status, and HIV status. The full sample included 967 OSGM and 15,563 non-OSGM. OSGM adults were younger (mean [SD], 63 [8] vs 66 [8], p<0.05), with higher frailty (36% vs 27%, p<0.05), similarly insured (98% vs 98%, p>0.05), and fewer reported “excellent” mental health (19% vs 26%, p<0.05). There were no significant associations between OSGM status and prescriptions in PIM categories of hypnotic/sedatives, antipsychotics, and antidepressants. However, OSGM status (aOR=1.79, 95% CI 1.09,2.79) and frailty (aOR=1.97, 95% CI 1.43,2.71) were associated with higher odds of anxiolytic PIM prescriptions. Our findings suggest that OSGM status and frailty were each independently associated with PIM anxiolytic prescriptions. This highlights a disparity in potentially inappropriate prescribing of anxiolytics among OSGM.

